# 735 Grill-Related Burns: A Matched Cohort Study

**DOI:** 10.1093/jbcr/irae036.278

**Published:** 2024-04-17

**Authors:** Deborah Choe, Michael N Cooper, Miki Roberts, Justin Gillenwater, Haig A Yenikomshian

**Affiliations:** Keck School of Medicine of USC, Los Angeles, CA; USC Plastic Surgery, Los Angeles, CA; Los Angeles General Hospital, Los Angeles, CA; Keck Medicine of USC, Los Angeles, CA; University of Southern California, Los Angeles, CA; Keck School of Medicine of USC, Los Angeles, CA; USC Plastic Surgery, Los Angeles, CA; Los Angeles General Hospital, Los Angeles, CA; Keck Medicine of USC, Los Angeles, CA; University of Southern California, Los Angeles, CA; Keck School of Medicine of USC, Los Angeles, CA; USC Plastic Surgery, Los Angeles, CA; Los Angeles General Hospital, Los Angeles, CA; Keck Medicine of USC, Los Angeles, CA; University of Southern California, Los Angeles, CA; Keck School of Medicine of USC, Los Angeles, CA; USC Plastic Surgery, Los Angeles, CA; Los Angeles General Hospital, Los Angeles, CA; Keck Medicine of USC, Los Angeles, CA; University of Southern California, Los Angeles, CA; Keck School of Medicine of USC, Los Angeles, CA; USC Plastic Surgery, Los Angeles, CA; Los Angeles General Hospital, Los Angeles, CA; Keck Medicine of USC, Los Angeles, CA; University of Southern California, Los Angeles, CA

## Abstract

**Introduction:**

Barbequing is a ubiquitous American pastime. However, unsafe practices involving this can result in devastating burn injury. Grills require a fuel source and an ignition which can be causes of injury. No US study to date has investigated the etiology of grill-related burns. This study aims to characterize the demographics, injury characteristics, and outcomes of grill-related burns and to identify ways of burn prevention.

**Methods:**

A retrospective review of patients admitted to a single-institution burn center from 1/1/17 - 7/1/23. Data included demographics, comorbidities, burn etiology, location, severity, hospitalization characteristics, and outcomes. Each grill cohort (G Cohort) patient was matched to a non-grill control by mBaux score and burn location. A random number generator was used when multiple controls met matching criteria.

**Results:**

Out of 2,355 patients, 69 (2.9%) met inclusion criteria. The G Cohort had 55 (79.7%) males and 5 (7.2%) pediatric patients. The average age was 41.7 ± 17.5 years old. A total of 20 (29.0%) grill-related burns occurred during Jun. - Aug. and 8 (11.6%) during Dec. - Feb. At time of admission, 25 (36.2%) patients had positive blood alcohol, 8 (11.6%) tested positive for amphetamine and 5 (7.2%) tested positive for cocaine. A total of 61 (88.4%) burns involved the upper extremity, 43 (62.3%) the head/neck, 34 (49.3%) the lower extremity, and 30 (43.5%) the trunk. A total of 24 (34.8%) burns were due to propane tank explosion, 18 (26.1%) related to lighter fluid use with 5 (7.2%) of them due to using gasoline or ethanol substitutes, 16 (23.2%) due to general flame injury, 9 (13.0%) due to contact burns, and 2 (2.9%) due to chemical burns related to grill cleaning solution. Compared to control, the G Cohort had greater proportions of male (79.7% vs. 62.3%, p< 0.05) and Hispanic/Latino patients (50.7% vs. 34.8%, p< 0.05) and a smaller proportion of Asians (2.9% vs. 10.1%, p< 0.05). The G Cohort had smaller proportions of patients who were undomiciled (11.6% vs. 30.4%, p< 0.01) or had a history of mental illness (5.8% vs. 17.4%, p< 0.05). Grill-related burns, which required fewer surgeries on average (1.4 ± 0.7 vs. 2.3 ± 1.9, p< 0.05), had a greater proportion of flash/flame burns (79.7% vs 55.1%, p< 0.01), with no differences in burn depth, hospital length of stay, ICU admission, readmission, or mortality rates.

**Conclusions:**

This study suggests that middle-aged, domiciled males without psychiatric comorbidities are more likely to make preventable grilling errors resulting in burn injuries. Prevention strategies targeting this demographic should underscore the risks of grilling while intoxicated, proper handling of propane tanks and lighter fluid, and the importance of using flash/flame-resistant gear protecting the upper extremities and head/neck.

**Applicability of Research to Practice:**

This study enhances our understanding of grill-related burns and ways to develop effective preventative strategies.

